# Physical micro-environment interventions for healthier eating in the workplace: protocol for a stepped wedge randomised controlled pilot trial

**DOI:** 10.1186/s40814-017-0141-z

**Published:** 2017-06-09

**Authors:** Milica Vasiljevic, Emma Cartwright, Rachel Pechey, Gareth J. Hollands, Dominique-Laurent Couturier, Susan A. Jebb, Theresa M. Marteau

**Affiliations:** 1 Behaviour and Health Research Unit, Institute of Public Health, University of Cambridge, Cambridge, UK; 2 Nuffield Department of Primary Care Health Sciences, University of Oxford, Oxford, UK

**Keywords:** Physical micro-environment interventions, Choice architecture, Nudging, Stepped wedge trial, Randomised controlled trial, Healthier eating, Workplace interventions, Size, Availability, Labelling

## Abstract

**Background:**

An estimated one third of energy is consumed in the workplace. The workplace is therefore an important context in which to reduce energy consumption to tackle the high rates of overweight and obesity in the general population. Altering environmental cues for food selection and consumption—physical micro-environment or ‘choice architecture’ interventions—has the potential to reduce energy intake. The first aim of this pilot trial is to estimate the potential impact upon energy purchased of three such environmental cues (size of portions, packages and tableware; availability of healthier vs. less healthy options; and energy labelling) in workplace cafeterias. A second aim of this pilot trial is to examine the feasibility of recruiting eligible worksites, and identify barriers to the feasibility and acceptability of implementing the interventions in preparation for a larger trial.

**Methods:**

Eighteen worksite cafeterias in England will be assigned to one of three intervention groups to assess the impact on energy purchased of altering (a) portion, package and tableware size (*n* = 6); (b) availability of healthier options (*n* = 6); and (c) energy (calorie) labelling (*n* = 6). Using a stepped wedge design, sites will implement allocated interventions at different time periods, as randomised.

**Discussion:**

This pilot trial will examine the feasibility of recruiting eligible worksites, and the feasibility and acceptability of implementing the interventions in preparation for a larger trial. In addition, a series of linear mixed models will be used to estimate the impact of each intervention on total energy (calories) purchased per time frame of analysis (daily or weekly) controlling for the total sales/transactions adjusted for calendar time and with random effects for worksite. These analyses will allow an estimate of an effect size of each of the three proposed interventions, which will form the basis of the sample size calculations necessary for a larger trial.

**Trial registration:**

ISRCTN52923504

## Background

Reducing excess consumption of food and drink is core to tackling the high prevalence of overweight and obesity in the population [1]. It is estimated that about one third of our daily energy intake is consumed while at work [2], with the majority of food consumed not brought from home [3]. The National Institute for Health and Care Excellence (NICE) estimates that a company employing 1000 people could lose more than £126,000 a year in reduced productivity solely due to obesity [4]. Whilst a systematic review is underway [5], there is currently limited evidence of the impact of interventions within workplaces to improve employees’ diets.

In a recent review that included six studies examining dietary interventions in the workplace, four reported small increases in fruit and vegetable consumption following implementation of a range of interventions [6]. However, outcomes were mainly based on self-report and incomplete reporting of all the studies made judgements of risk of bias unclear. In addition, the range of interventions assessed was based largely on providing information (with most studies implementing different kinds of nutritional education campaigns via posters, leaflets, and group workshops delivered at workplaces), commonly regarded as insufficient to tackle obesity [7]. Evidence is now accumulating to suggest that altering cues in small-scale or micro physical environments may provide a more effective way of changing behaviour, often without awareness and in ways that do not place demands on our limited cognitive resources [8, 9]. This is sometimes referred to as ‘nudging’ or ‘choice architecture’ [10]. In keeping with this, a recent report on obesity identified the potential value of a number of such choice architecture interventions [7].

We propose a pilot study to estimate the potential impact of three sets of physical micro-environment interventions to reduce energy purchased in workplace cafeterias in preparation for a future larger trial. A further aim is to examine the feasibility of recruiting eligible worksites, and identify barriers to the feasibility and acceptability of implementing the interventions in preparation for a larger trial.

### Portion, package and tableware size

One promising target for interventions in physical micro-environments is the size of food portions, their packages and the tableware used to consume them. The most robust review to date of the impact of portion, package and tableware size upon consumption [11, 12], suggests that the scale of the ‘portion size effect’ (describing the observation that people consistently consume more when offered larger portions, packages or tableware) is such that, removing larger portions, packages and tableware from the diet could reduce the daily energy intake of UK adults by up to 16% (279 calories).

### Availability of healthier vs. less healthy foods

A second intervention is to alter the availability of foods and drinks by increasing the range of healthier foods/drink options and/or decreasing the range of less healthy options. A recent review of interventions in vending machines found that sales of healthier items were increased in five of the six identified studies that increased their availability, with no loss of overall sales volume [13]. Indeed, availability and portion size together represent two of the top three interventions suggested in the McKinsey Global Institute report [7] as having the highest likely impact across the population. A Cochrane review of the impact of availability interventions in all settings is currently underway [14].

### Energy labelling

A third intervention that can be used in workplace settings is to provide labels displaying energy (calorie) content. Recent review evidence shows that calorie labelling at point of consumption can reduce average daily energy intake from food and drinks [15].

However, recent Cochrane reviews of the three interventions used in this pilot study (11, 14, 15) have not identified any randomised studies in worksite cafeterias. Prior to designing and conducting a larger trial, several key design uncertainties need to be addressed. These include the feasibility of recruiting eligible worksites, the suitability of workplaces for implementing one of the three interventions, the practicalities associated with implementing the interventions including any challenges with implementation or acceptability, and worksites’ ability to provide the required data and comply with the study protocol. The pilot study will also enable us to estimate required sample sizes needed for a larger trial, by providing estimates of likely effect sizes for each of the three interventions and identifying key characteristics and issues with the data that are likely to be encountered.

We propose to pilot three types of interventions with the potential to reduce the energy from food and drink purchased in workplace cafeterias:

i. reducing the size of portions, packages and tableware;
ii. increasing the availability of lower energy (calorie) foods and drinks, relative to higher energy foods and drinks;
iii. labelling foods and drinks with their energy (calorie) content.

In particular, we will examine:

- The impact upon the total energy (calories) of food and drink purchased in workplace cafeterias of:
- smaller portions and packages of foods and drinks, and smaller tableware;
- removing some of the higher energy items available and replacing these with lower energy options;
- providing energy (calorie) labels on foods and drinks.
- The extent to which the impact of the interventions varies with the socio-demographic profiles of the participating workplaces.

### Interventions

These will take place in worksite cafeterias. The precise nature of the interventions will vary between sites, depending upon the food, drink and tableware currently available. For example, if sugar-sweetened beverages are already those at the smallest available size (e.g. a 150 ml can or carton of drink) these will not be a focus for reduction in size. The target products for each intervention will be documented and included as covariates in the analyses.

**Fig. 1:**
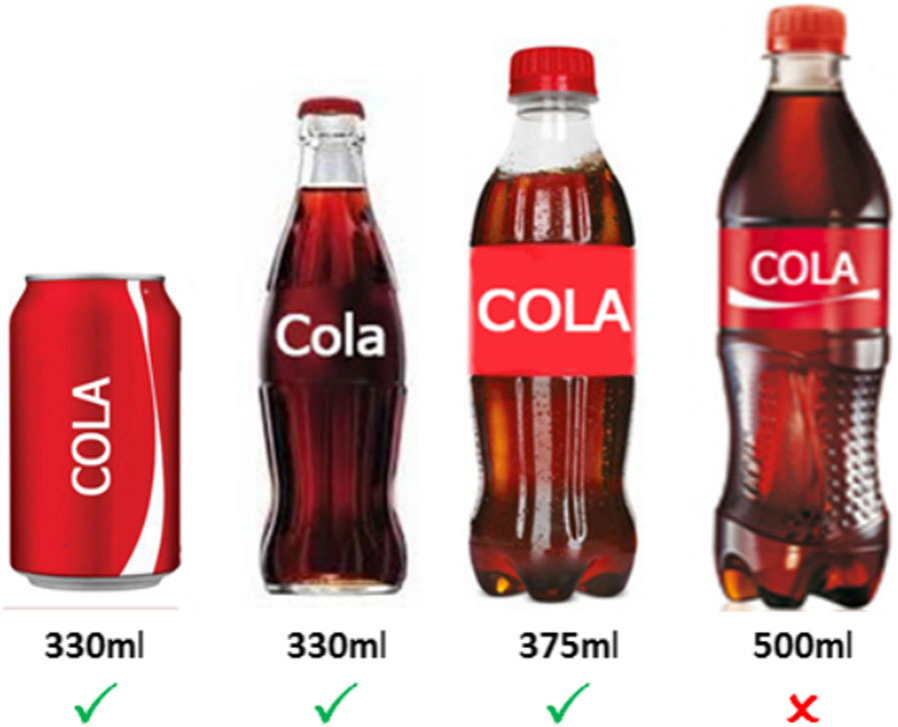
An example of intervening on the size of packaged products (e.g. replacing a 500 ml bottle with a 375 ml bottle or 330 ml bottle or can)
  - *Tableware*: the size of available glasses, plates, bowls and/or cutlery used to serve and consume food and drink items will be reduced to the next smaller available size. For example, a plate 30 cm in diameter may be replaced by a plate 27 cm in diameter.
  For all sites, the standard retail price of the packages and portions will be used. In the case of reducing portion size of items which are not pre-packaged (such as main meals or cakes), prices will be reduced proportionally.
ii. Availability
  Research question
  What is the impact on energy purchased of reducing the number of less healthy foods and drinks available in worksite cafeterias and replacing these with the equivalent number of healthier food and drinks?
  This intervention will involve keeping constant the number of options offered but altering the ratio of healthier to less healthy options by reducing the less healthy foods and drinks (products or units of the same product) available and increasing the healthier foods and drinks available (see Fig. 2 for an example).

In particular, for the size intervention we propose to intervene on all products sold in the workplace cafeterias for which the energy content can be meaningfully reduced. We will therefore exclude from the size intervention products containing little or no energy (<5 kcal/100 g), such as water or chewing gum, and all fresh fruit and vegetables with no added fat or sugar. For the availability intervention, we propose using predominantly energy-based cut-offs to define ‘healthier’ vs. ‘less healthy’ options (more details are provided below under the availability intervention description). For the labelling intervention, we propose to intervene on all products sold in the workplace regardless of macronutrient profile (with the exception of items made individually by the workplace patrons for which the energy content of individually made items cannot be determined beforehand, for example for foods offered on a salad or a wok bar). For all three interventions, hot drinks will also be excluded, due to difficulties with recording the sales data for these products across worksites.

i. Size
  Research question
  What is the impact on energy purchased of reducing the size of portions and/or packages of foods and drinks and/or tableware size in worksite cafeterias?
  The intervention will be applied across a combination of the following:
  - *Portion sizes of served foods*: reduce the size of portions of higher energy food and drink items served in worksite cafeterias by approximately 10% to 15%, including
    Main meals (e.g. lasagne)
    Sides (e.g. chips or fries)
    Desserts, cakes, cookies and biscuits
    This might involve producing 16 portions of lasagne from a tray that was previously cut into 14 portions, or 14 slices of cake from a cake that was previously cut into 12 slices.
  - *Pre-packaged products*: replace currently available packaged food and drinks in cafeterias with the next smaller available package size (see Fig. 1 for an example).

**Fig. 2:**
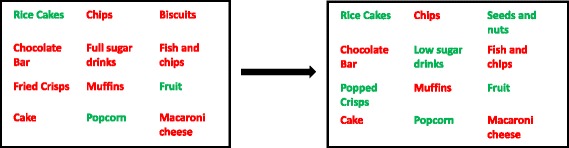
A graphical presentation of the availability intervention (e.g. replacing a proportion of higher energy options with lower energy ones)
  The size of the change will depend upon the baseline ratio of healthier to less healthy products. For example, in worksite cafeterias where 75% of available options are classified as less healthy the intervention would likely involve reducing this to 50% of options being less healthy.
  The terms ‘healthier’ and ‘less healthy’ are used to reflect that these products are either side of a point in a continuum of healthiness, rather than ‘healthy’ and ‘unhealthy’ which might suggest taking products from either extreme (for more details see Table 1). Using only products that are at the extremes of the healthiness continuum would limit the range of items across which the intervention could be applied.

**Table 1:** Example definition of ‘healthier’ vs. ‘less healthy’ options for the availability intervention

Categories	Items	Cut-off	Higher energy	Lower energy
Cooked main meal (excluding breakfast)	Complete main meals	500+ Cals ^a^	e.g. beef lasagne, chilli con carne and rice	e.g. salads, vegetable tagine and couscous
	Main with side to add	300+ Cals ^a^	e.g. hamburgers, battered fish	e.g. vegetable quiche, chicken breast filled with ricotta
	Sides	Added fat	Potatoes: Roast, saute, wedges, chips, mash; Garlic bread; Onion rings; Onion bhaji	Potatoes: Boiled, baked. Rice, couscous
Sandwiches or equivalents	Sandwiches, wraps, panini, baguettes, bagels, pasties	350+ Cals ^a^		
Snacks	Savoury snacks	120+ Cals	e.g. full-fat, baked or vegetable crisps	e.g. popped crisps, popcorn, coconut curls
	Sweet snacks	150+ Cals	e.g. standard chocolate bars, biscuits	e.g. selected cereal bars (Nakd), popcorn bars, rice cakes, lower calorie (small) chocolate bars: Milky Way, Freddo, Fudge
Drinks	Cold drinks (100% fruit juice and smoothies excluded, up to 250 ml bottles)	50+ Cals	e.g. full-sugar soda, flavoured water, juice-based drinks, squashes, energy drinks	e.g. water, diet soda, lower calorie drinks (Vit Hit etc)
Soups		Added cream	Cream based	Broth or tomato based
Dessert	Dessert pot/ice cream	200+ Cals	e.g. mousse, trifle, ice cream	e.g. yoghurt
	Cakes	200+ Cals	e.g. muffins, cupcakes, sponge sandwich	e.g. teacake, fruit loaf
	Served desserts	200+ Cals	e.g. crumbles, pies, fruit tarts	e.g. low-calorie jelly, sorbet

^a^ Based on Change 4 Life recommending a 400 – 600 –600 (Breakfast – Lunch – Dinner) calorie split (leaving some calories for drinks/snacks)

Lunch: With the 600 calorie allowance:

- Allow 100 calories for vegetables

- Allow 200 calories for potato sides

**Fig. 3:**
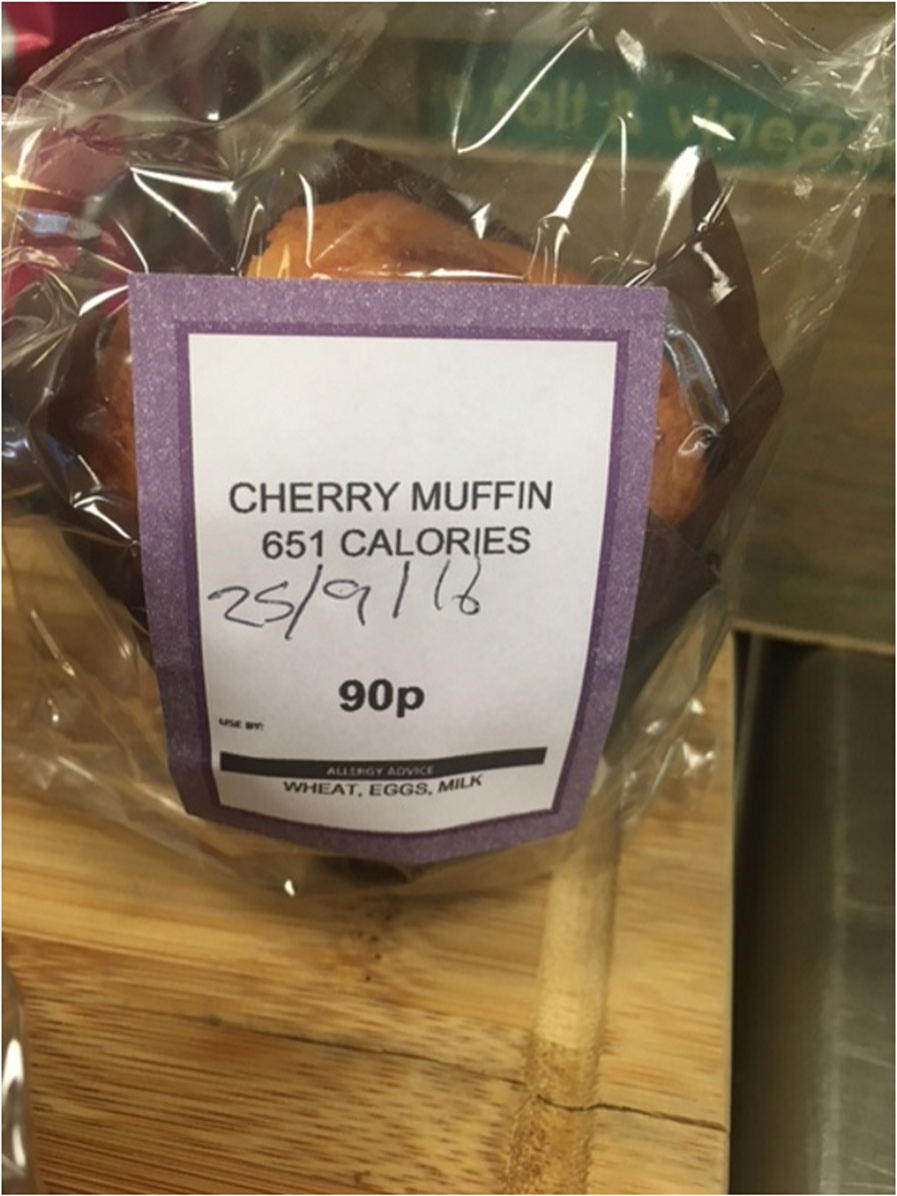
An example of a product displaying energy labelling

So, for meals that are ‘all-in-one’ (or have a standard accompaniment that can be rolled into the calorie count, e.g. chilli and rice), less healthy cut-off is 500 calories. For meals that come with potato side, cut-off is 300 calories
iii. Labelling
  Research question
  What is the impact on energy purchased of adding labels showing energy (calorie) content on items purchased from worksite cafeterias?
  This intervention comprises labels that state the name of a food or drink item together with the energy content written as ‘XXX Calories’. The latter is to be written in the same font type and size as the product name and/or price information, legible and prominent to the customer, rounded to the nearest 5 or 10 calories, and clearly denoting the portion size to which it refers (see Fig. 3 for an example). In keeping with EU regulations, kilocalorie (kcal) and kilojoule (kj) content will be displayed beneath the ‘XXX Calories’.

## Methods

### Study design

**Fig. 4:**
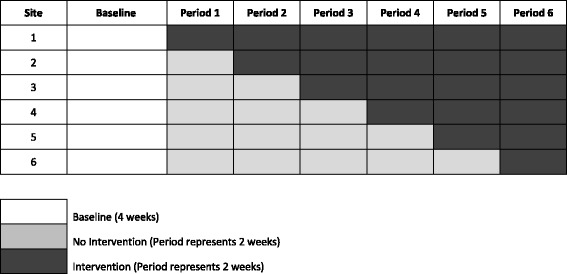
A graphical presentation of the stepped wedge design used for each intervention in the study

A stepped wedge design [16] (see Fig. 4) was selected, due to being simpler to implement and better able to capture intervention effects than parallel cluster randomised or multiple-treatment reversal (ABAB) designs. Stepped wedge trials are a type of RCT which involve sequential, but random rollout of an intervention over multiple time periods. Stepped wedge trials typically have a baseline period in which observations are made while all participating sites are unexposed to the intervention, and one time period in which all participating sites are exposed to the intervention (at the end of the trial) [16]. As such, this design allows examination of changes in purchasing within each worksite cafeteria depending on the time-period (baseline vs. intervention); as well as differences in purchasing between worksite cafeterias at different time-points of the stepped wedge trial. Each intervention will be evaluated singly, in one of three sets of six worksites. The 18 worksite cafeterias (one cafeteria per worksite) will be allocated to one of these three sets depending on their readiness to start, determined by data collection systems in place. The first six sites to be ready will be allocated to the first intervention ready for implementation: labelling. The remaining 12 sites will be randomly allocated to implement either the size or the availability interventions. The unit of analyses is the worksite cafeteria, not the individuals using the worksite cafeteria (see Fig. 4).

Within each of the three sets of six worksite cafeterias, the time at which the intervention is introduced will be randomly determined to control for time trends while maximising sample size. Worksite cafeterias will be allocated to a phase of the stepped wedge design by means of random permutations using random variates of the uniform distribution [17]. The randomisation will be performed by a statistician, with the assistance of computer software.

### Sample

Eighteen English worksite cafeterias recruited from companies that are members of the Institute for Grocery Distribution (IGD; comprising 1027 members), selected on the basis of:

1. Number of employees (>350)
2. Mix of office-based and other site types (to recruit sites that are likely to represent different ranges of socio-economic status)
3. Ability to provide weekly data on sales of individual items and their energy content

**Fig. 5:**
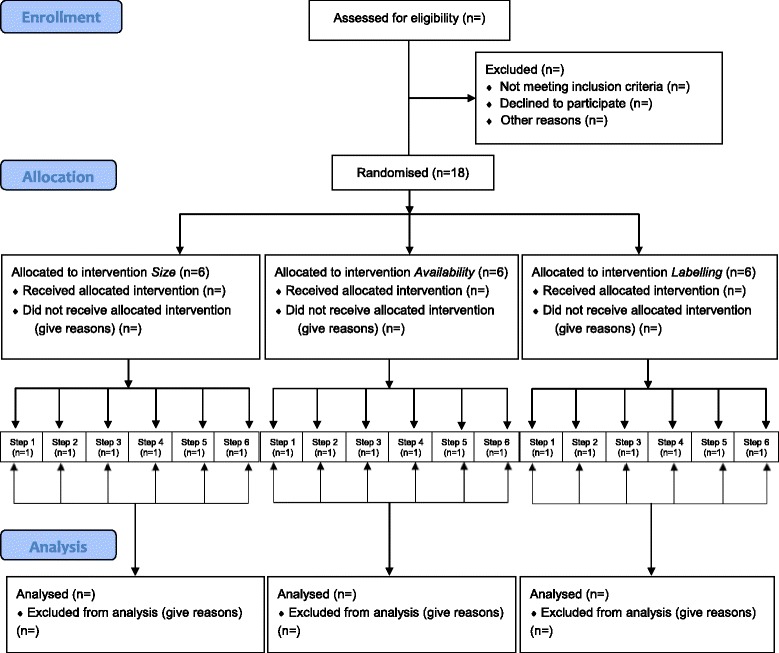
CONSORT flow diagram

Worksites from any region within England would be eligible. Managers of identified eligible worksites will be contacted, and given details on the design of the pilot trial, as well as all the requirements regarding participation in the pilot trial, including the range of possible timings for implementing the intervention in the stepped wedge design. Please see Fig. 5 for a CONSORT flow diagram delineating the flow of participating sites through the pilot trial.

### Measures

#### Feasibility assessment measures

1. *Feasibility of recruiting and retaining eligible worksites:* measured by recording recruitment rates, and the number of worksite cafeterias dropping-out of the pilot trial (i) post-recruitment, (ii) during the baseline or (iii) during the intervention period.
2. *Feasibility of implementing the assigned intervention:* determined after initial visits to worksite cafeterias by the research team, discussions regarding suitability with worksite managers and catering teams, and by examining the sales data supplied by the sites. Qualitative interviews with worksite managers will provide an additional measure of potential challenges with implementation of the intervention.
3. *Acceptability of the interventions:* measured by surveying patrons of the worksite cafeterias regarding acceptability of, and other perceptions concerning, the intervention. Qualitative interviews with worksite managers will supplement the survey data, providing insight into the acceptability of study and assessment procedures.
4. *Compliance with the study protocol:* assessed via compliance visits coinciding with the initial period of intervention implementation for each randomised worksite.

### Intervention impact measures

#### Primary outcome

Total energy (calories) purchased per time frame of analysis controlling for the total sales/transactions.

#### Secondary outcome

Total energy (calories) purchased per time frame of analysis from (a) intervention items, and (b) non-intervention items controlling for the total sales/transactions.

#### Other measures

Various other measures will be collected that will allow us to estimate the primary and secondary outcomes with greater precision. Covariates examined for all three interventions include: worksite demographic characteristics (age, gender, and dominant occupational status); day of week (if the distribution of the data allows for analysis per day); and weather conditions.

The following will also be recorded for each of the three interventions:

i) Size: mean energy (calorie) per product pre-intervention; mean reduction (%) in energy (calorie) per product; mean price pre-intervention; mean reduction (%) in price; mean size of tableware pre-intervention; mean reduction (%) in size of tableware.
ii) Availability: proportion of food/drink intervention items that are healthier pre-intervention; proportion of food/drink items that are targeted in the intervention; mean energy (calories) per item pre-intervention; reduction (%) in proportion of intervention items that are healthier from pre-intervention; reduction (%) in energy (calories) per item from pre-intervention; Mean price pre-intervention; mean change (%) in price.
iii) Labelling: mean energy (calories) per item pre-intervention; mean energy (calories) per item during intervention.

### Planned analyses

#### Feasibility assessment analyses

Feasibility and acceptability outcomes, including recruitment and attrition rates, will be reported using descriptive statistics. Qualitative assessments gathered via semi-structured interviews with worksite cafeteria staff will be coded and summarised in narrative form.

#### Intervention impact analyses

One of the main aims of this pilot trial is to estimate the effect size of each of the three interventions, to inform the sample size calculations necessary for a future, larger trial. This will be achieved by conducting quantitative analyses using linear mixed models, which will be reported in full in the scientific paper reporting the results of the pilot trial. By estimating intervention parameters, while taking into account the within-site data dependence and controlling for other parameters, mixed models will help define the effect size of each intervention.

The potential impact of each intervention will be estimated in separate linear mixed models examining the impact on total energy (calories) purchased per time frame of analysis controlling for the total sales/transactions, adjusted for calendar time and with random effects for worksite.

If heteroscedasticity due to different worksite sizes (number of employees) is observed and/or if model assumptions are not respected, outcome change of scale would be considered, as well as alternative analyses considering, for example, weighting worksites according to their variance [18]. If mixed model assumptions are not met, generalised estimating equations (GEE) will be preferred.

Initial analyses will be conducted to confirm the selected time frame level of weekly (as opposed to daily) sales. A weekly time frame will be selected if the conditional distribution of the daily outcome is incompatible with the model assumptions (after transformation), and if time-dependence is still present after controlling for total sales/transactions.

#### Other analyses

Other analyses will be conducted to inform the design of the main study. These analyses include attempting to estimate the intra-cluster correlation (ICC) using aggregated data, as parallel cluster randomised trials are known to be more efficient than stepped wedge design trials when the ICC is small [16].

### Procedure

For each of the three interventions, all six worksite cafeterias will initially undergo a baseline period (4 weeks) in which their usual sales are recorded. Sites will then be randomised to implement the intervention at one of six, two-weekly intervals.

## Discussion

The current pilot study is designed to finalise the design and conduct of a future, full-scale trial to assess the impact of three sets of physical micro-environment interventions with the potential to reduce energy consumption in the workplace. As well as providing exploratory estimates of the effect sizes of each of the three interventions, this pilot study will address key design uncertainties for the trial including the feasibility of recruiting eligible worksites, the suitability of workplaces for implementing one of the three interventions, the practicalities associated with implementing the interventions including any challenges with implementation, worksites’ ability to provide the required data and to comply with the study design. The pilot study will also allow us to explore the acceptability of the study procedures and assessment methods.

In addition to the strengths described above, this pilot trial has several limitations. First, the outcome measures pertain to purchasing and not consumption of energy. Although purchasing provides an indication of consumption in the workplace, it does not take into account food obtained from other sources or food waste. Second, this study will not directly measure change in the purchasing of individual workers, since the units of randomisation and analysis are the individual worksite cafeterias. Finally, this study measures changes in purchasing over a relatively short time-period; any longer-term impact of the interventions on worksite cafeterias (or the employees using them) will not be assessed. This pilot trial will nevertheless provide an indication of whether a longer follow-up in the larger trial is feasible, as well as the possibility of supplementing the main purchasing measures with complementary self-report measures of employees’ diets over time.

The stepped wedge design has strengths and limitations. A strength of this design is that it combines features of within- and between-subjects designs thus allowing the examination of changes in purchasing within each worksite cafeteria depending on the time-period (baseline vs. intervention). It also allows examination of differences in purchasing between worksite cafeterias at different time-points. Furthermore, in the current context, the stepped wedge design is expected to have more statistical power than a parallel group design. It is also more suitable than a multiple-treatment reversal (ABAB) design, which would alternate intervention and intervention free periods, as the effect of an intervention may extend beyond the strict intervention time frame. A notable limitation of a stepped wedge design is the potential for an underlying temporal trend to confound the intervention effect given more participating units are exposed to the intervention at later than earlier time-periods [19].

This pilot trial will inform a future larger trial that is expected to extend current knowledge regarding the effectiveness of physical micro-environment interventions (size, availability, and labelling), applied singly and together, to reduce the energy purchased in worksite cafeterias.
